# 

**Published:** 1919-12

**Authors:** 


					CONTENTS. page
Contemporary Medicine from the Standpoint of Pathology.
By
I. Walker IIall, M.D.
i?5
Disease in Mesopotamia.
By Major F. P. Mackie, M.D., M.Sc.,
F.R.C.S., F.R.C.P., I.M.S. ...
n8
On the Treatment of Ununited Fractures, with Especial Reference
to the Use of Bone Grafts.
By Ernest W. Hey Groves, M.S.,
F.R.C.S.
132
Stammering as it Occurred in the War.
By R. G. Gordon, M.D.,
B.Sc., M.R.C.P. Ed. ..
143
^ogress of the Medical Sciences
i5i
Reviews of Books
If>3
The Library of the Bristol Medico-Chirurgical Society   171
Meetings of Societies
174
Obituary Notices :
Nelson Congreve Dobson
177
Hugh Champneys Thurston
182
Sir William Osler ...
i83
Local Medical Notes  -   183

				

